# Synchronous gallbladder adenocarcinoma and gastric gastrointestinal stromal tumor: Case report and literature review

**DOI:** 10.1016/j.ijscr.2019.02.001

**Published:** 2019-02-13

**Authors:** Paulo Henrique Peitl Gregório, Lucas Seiti Takemura, André Luiz Vilela Galvão, Giuliano Campolim Gagliotti, Rodrigo Camargo Leão Edelmuth, Vanderlei Segatelli

**Affiliations:** a Division of General Surgery, Hospital Israelita Albert Einstein, São Paulo, Brazil; b Division of Pathology, Hospital Israelita Albert Einstein, São Paulo, Brazil

**Keywords:** Gastrointestinal stromal tumor, Gallbladder adenocarcinoma, Synchronous neoplasms, Case report

## Abstract

**Introduction:**

Synchronous occurrence of different types of neoplasms is not very frequent, representing around 6% of all cases of cancer. Usually there is a lack of information on how to treat these patients, especially when both types of cancers are also uncommon. No cases of synchronous gallbladder adenocarcinoma and gastric gastrointestinal stromal tumor have been published before.

**Presentation of case:**

We present the case, management and follow-up, of a 66-year-old female with incidental diagnosis of a pT2NxMx gallbladder adenocarcinoma after elective cholecystectomy that latter, during staging, was also diagnosed with GIST. Total gastrectomy, wedge resection of the liver and lymphadenectomy were performed due to the new findings. Adjuvant chemotherapy for 36 months was indicated. After 16 months of the treatment she has no signs of recurrence.

**Discussion:**

Gastrointestinal stromal tumors (GISTs) had a turnaround in the end of the 20th century after the introduction tyrosine-kinase inhibitor to the adjuvant treatment and now the trend is to extend it up to 36 months in selected patients. Gallbladder adenocarcinoma is an uncommon cancer but the incidental diagnosis is increasing with the popularity of laparoscopic cholecystectomy and, thus, specific management should be offered for these patients, what frequently includes a complementary surgery. Although, GISTs may be associated with another synchronous tumor in 20% of the cases, the simultaneous occurrence with gallbladder cancer is incredibly rare.

**Conclusion:**

Simultaneous occurrence of gastric GIST and gallbladder adenocarcinoma has not been reported before and, thus, any information about it may help in the management of those patients.

## Highlights

• First report of synchronous incidental gallbladder adenocarcinoma and gastric GIST.
• Incidental diagnosis of gallbladder cancer with GIST diagnosed during staging.
• Patient is under adjuvant treatment and after 16months has no signs of recurrence.

## 1 Introduction

Simultaneous primary tumors have been reported for a long time. In 1921, Owen showed 4.7% of cases of multiple tumors in a 3000 case-series [1]. In 2009, a European study registered 2.919.023 cases of malignant cancers and reported a total of 183.683 multiple primary tumors (synchronous and metachronous), representing 6,3% of the overall evaluated [2]. The simultaneous occurrence of two or more tumors have a profound impact on the treatment approach since the ideal anticancer therapy would have to be effective against both. These patients are usually excluded from clinical research protocols and consequently, there is a lack of superior quality evidence on how to treat them. Their survival rates are, commonly, lower when compared to single primary tumor populations [2,3].

Gallbladder adenocarcinoma and gastric gastrointestinal stromal tumor (GIST) are both uncommon types of cancer and its simultaneous occurrence has not been reported previously. We report a case of a woman with incidental diagnosis of gallbladder adenocarcinoma after elective cholecystectomy with a subsequent diagnosis of GIST during the staging.

Patient has signed an informed consent and agrees with the following article in its entirety, also, this publication was approved by a Local Research Committee, is registered in the national research database and has been reported according to SCARE criteria [4].

## 2 Case-report

A 66-year-old black woman was submitted to elective laparoscopic cholecystectomy on August 2016, when she had asymptomatic cholelithiasis diagnosed on a routine abdominal ultrasound. Her past medical history included just arterial systemic hypertension with irregular treatment. She denied any other comorbidities and did not have any previous surgeries. The surgery and her recovery were uneventful.

**Fig. 1:**
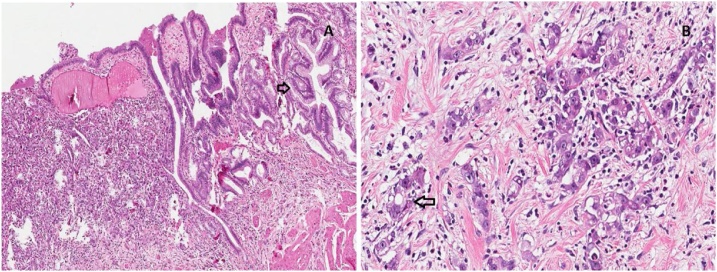
Gallbladder adenocarcinoma: (A) Mucosa with intestinal metaplasia(arrow) and architecture destruction (left side) caused by infiltrative epithelial neoplasia (H&E 10×); (B) Infiltrative neoplasia with large nucleus, evident nucleolus and creating tubular structures (arrow) (H&E 20×).

The pathological analysis of the gallbladder revealed an infiltrative neoplasia with large nucleus causing architectural destruction and intestinal metaplasia. It was reported as an adenocarcinoma of 1,1 cm with free margins, subserosal invasion, with no lymphatic or perineural involvement, staging pT2NxMx (Fig. 1).

**Fig. 2:**
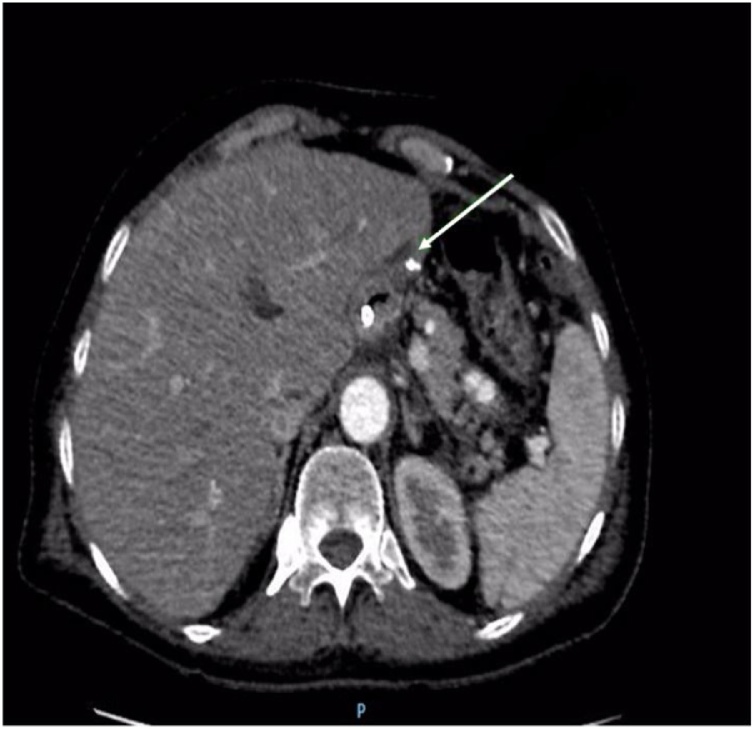
Abdomen CT for staging of gallbladder adenocarcinoma: Wall thickening in the proximal gastric segment with exophytic component close to the lesser curvature, measuring 5 × 4 cm (arrow).

In October 2016 a staging abdominal computerized tomography(CT) was performed and showed irregular wall thickening in the proximal gastric segment with exophytic component close to the lesser curvature, measuring 5 × 4,5 cm (Fig. 2). No hepatic lesions or lymphadenopathies were found.

**Fig. 3:**
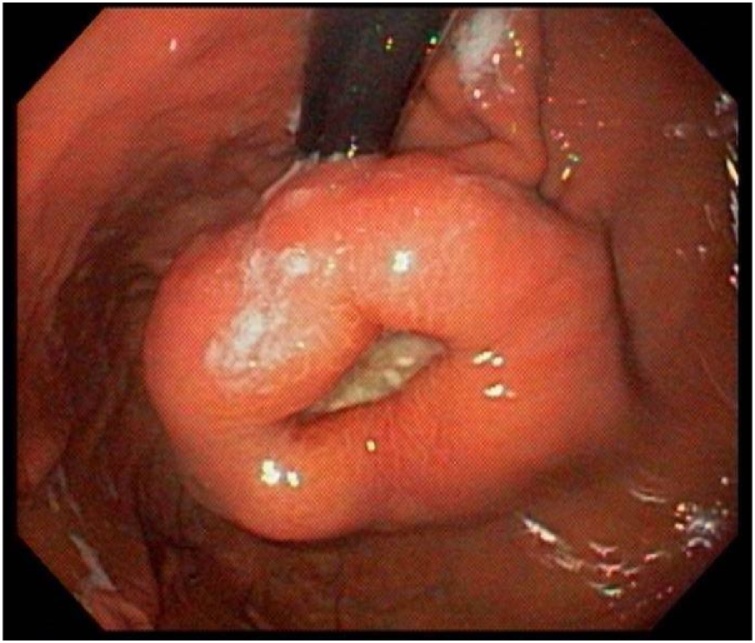
Upper gastrointestinal endoscopy: CT finding of an exophytic mass in the gastric small curvature was corroborated by further endoscopical evaluation.

**Fig. 4:**
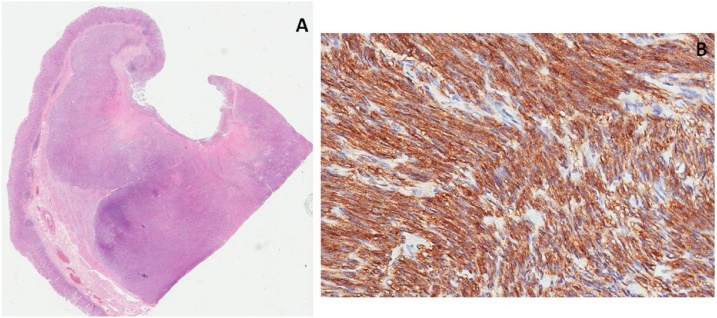
Histopathological analysis of gastric lesion: (A) Mucous ulceration by expansive growth of the gastric wall neoplasm (H&E 20×); (B) Neoplastic cells with positive immunoexpression of CD117/c-*kit* (H&E 20×).

One week later an upper gastrointestinal endoscopy found a submucous lesion 3 cm below the cardia, with central ulceration and indurated edges (Fig. 3). Histopathologic analysis of the ulcerated lesion showed mucous ulceration by expansive growth of the gastric wall neoplasm with positivity to CD117, CD34 and DOG-1 and negativity to S100, Desmin, AE1AE3 and smooth-muscle actin (Fig. 4).

On November 2016, total gastrectomy with Roux-en-Y anastomosis, wedge resection of the gallbladder bed (resection of IVb and V liver segments) and hepatic hilar lymphadenectomy were executed. Pathological analysis revealed a 4,5 × 3,5 cm GIST with 12 mitosis in 5 mm² (20 high power fields), high histologic grades (G2) and free margins. Thirty lymph nodes were evaluated, and none showed signs of malignancy. Liver segments were not compromised. GIST was staged as a pT2N0M0. The patient was classified as high-risk of recurrence since the mitotic count was greater than 10/50 high power fields (NIH modified classification) and, thus, adjuvant chemotherapy was considered beneficial for her.

After the surgery, the patient developed esophagojejunal fistula which was managed conservatively. She also had asymptomatic pulmonary thromboembolism in the right descendent interlobar artery, which was treated with anticoagulation.

On February 2017 she started a 36 months treatment with Imatinib mesylate (Gleevec/Glivec, Novartis, Basel, Switzerland) 400 mg daily and so far, after 16 months follow-up, she has no signs of recurrence, with CEA, CA19-9 and abdominal/chest CTs with no abnormalities.

## 3 Discussion

Gastrointestinal stromal tumors (GISTs) had a turnaround in 1998 when it was identified that the gain-of-function mutations of the c-*kit* gene have an important role in the oncogenesis of GISTs with more than 95% of GISTs expressing c-*kit* [5]. The first use of Imatinib, a tyrosine-kinase inhibitor, 3 years later as adjuvant therapy in GIST was a milestone on the disease treatment and its approval as the main therapy for GIST happened in 2008 [6]. The use of adjuvant therapy today is based on the risk-stratification schemes such as National Institute of Health(NIH) consensus criteria, NIH-modified consensus criteria and Armed Forces Institute of Pathology(AFIP) criteria. In 2012, Joensuu et al. compared the prognostic accuracy between those schemes using receiver operating characteristic (ROC) analysis. The area under the curve when estimating the 10-year risk of GIST recurrence were similar for the three schemes: NIH consensus classification criteria = 0.79; NIH modified consensus classification criteria = 0.78; AFIP criteria = 0.82. Moreover, NIH-modified consensus criteria, when evaluating recurrence-free survival(RFS) vs. time from diagnosis (years), identified a subgroup of high-risk patients that has more benefit to receive adjuvant therapy due to unfavorable prognosis [7].

Nowadays, GIST is an atypical case of solid tumor which receives adjuvant therapy for more than one year. In 2012, a multicentric research conducted in four European countries allocated equally 400 patients between a group that received Imatinib for 12 months and another group that received it for 36 months. Individuals who received 36months had greater RFS (hazard ratio [HR], 0.46; P < .001; 5-year RFS, 65.6% vs 47.9%) and longer overall survival (HR, 0.45; 95%; P = .02; 5-year survival, 92.0% vs 81.7%). [8]. While three years of adjuvant imatinib is the standard for patients with estimated high-risk of recurrence, a developing clinical trial (PERSIST-5 / NCT00867113) shows that a five-year therapy would reduce recurrence in patients with sensitive mutations and that most recurrences would follow imatinib discontinuation [9].

Gallbladder adenocarcinoma is also rare and ranks sixth for all gastrointestinal tumors. It has an important geographical variation, with higher incidence rates among American and South American Indians [10]. It is an aggressive cancer with the shortest median survival from the time of diagnosis to death and adenocarcinoma represents more than 95% of the cases [10,11]. The majority of patients are asymptomatic and diagnosis is made either intraoperatively or on pathological analysis after elective cholecystectomy [12]. A 2011 study shows that 26% of incidental T2 tumors have liver involvement, what is associated with lower RFS and disease-specific survival (*p* = 0.004 and p = 0.003 respectively) when compared to T2 tumor without liver involvement [13]. Currently, National Comprehensive Cancer Network^®^ and European Society for Medical Oncology recommend that all postoperative incidentally diagnosed gallbladder cancers above T1a should do gallbladder bed resection and lymphadenectomy including porta hepatis, gastrohepatic ligament and retroduodenal lymph nodes since simple cholecystectomy has a 5-year survival rate of only 20–40% [14].

Several reports evaluating the simultaneous occurrence of GISTs and synchronous gastrointestinal neoplasms have been released over the last decade. Approximately 10–20% of patients with GISTs have a second primary tumor [15]. The first study that evaluated this fact in a population-level was released in 2015 using the database of Surveillance, Epidemiology and End Results (SEER), which analyzed 6.112 GIST cases over a period of 10 years. They found that 1.047 patients (17.1%) had additional cancers associated with GIST specially in the genitourinary (35.8%) and gastrointestinal tract (17.2%). The latter was mainly due to colorectal cancer. This study provided evidence for an increased risk of cancer among the GIST population [15], but until the moment this case report was submitted we could not find any case of synchronous gastric GIST and gallbladder adenocarcinoma published in english over MedLine database.

In summary, patients with GIST may have unknown pathogenic mechanisms that may increase the risk of developing other types of cancer. The proper identification and description of these links carry immense clinical implications, from screening and prevention to diagnosis and treatment. Due to the rarity of GIST and gallbladder adenocarcinoma synchronous occurrence, its evaluation and management become very challenging.

## Conflicts of interest

The authors do not have any conflict of interest in this research.

## Sources of funding

This study did not have a funding source.

## Ethical approval

Local Research Committee of Hospital Israelita Albert Einstein approved the publication of this case report under the reference number 2.946.926. The study has been also registered in the national research database (Plataforma Brasil).

## Consent

The patient agreed and signed an informed consent allowing the publication of his data including radiology and pathology images.

## Author’s contribution

Paulo Henrique Peitl Gregório: Study concept, writing paper, data collection and outpatient clinic consultation.

Lucas Seiti Takemura: Writing paper, literature review and data collection.

André Luiz Vilela Galvão: Writing paper, literature review and data collection.

Giuliano Campolim Gagliotti: Surgeon in both procedures, outpatient clinic consultation and data collection.

Rodrigo Camargo Leão Edelmuth: Surgeon in both procedures, outpatient clinic consultation and data collection.

Vanderlei Segatelli: Pathological analyzis and photography of specimens and paper reviewer.

## Registration of research studies

This study has been registered in the national research database (Plataforma Brasil).

Registration number (CAAE):99493018.3.0000.0071.

## Guarantor

Paulo Henrique Peitl Gregório - Division of General Surgery, Hospital Israelita Albert Einstein, São Paulo, Brazil.

## Provenance and peer review

Not commissioned, externally peer-reviewed.
